# Effect of Early Pelvic Binder Use in the Emergency Management of Suspected Pelvic Trauma: A Retrospective Cohort Study

**DOI:** 10.3390/ijerph14101217

**Published:** 2017-10-12

**Authors:** Sheng-Der Hsu, Cheng-Jueng Chen, Yu-Ching Chou, Sheng-Hao Wang, De-Chuan Chan

**Affiliations:** 1 Division of Traumatic Surgery, Department of Surgery, Tri-Service General Hospital, National Defense Medical Center, Taipei 11486, Taiwan; 2 Division of General Surgery, Department of Surgery, Tri-Service General Hospital, National Defense Medical Center, Taipei 11486, Taiwan; 3 School of Public Health, National Defense Medical Center, Taipei 11490, Taiwan; 4 Division of Orthopedic Surgery, Tri-Service General Hospital, National Defense Medical Center, Taipei 11486, Taiwan

**Keywords:** trauma, pelvic fracture, pelvic binder, external fixation, management

## Abstract

Background: We aimed to evaluate the effect of early pelvic binder use in the emergency management of suspected pelvic trauma, compared with the conventional stepwise approach. Methods: We enrolled trauma patients with initial stabilization using a pelvic binder when suspecting pelvic injury. The inclusion criteria were traumatic injury requiring a trauma team and at least one of the following: a loss of consciousness or a Glasgow coma score (GCS) of <13; systolic blood pressure of <90 mmHg; falling from ≥6 m; injury to multiple vital organs; and suspected pelvic injury. Various parameters, including gender, age, mechanism of injury, GCS, mortality, hospital stay, initial vital signs, revised trauma score, injury severity score, and outcome, were assessed and compared with historical controls. Results: A total of 204 patients with high-energy multiple-trauma from a single level I trauma center in North Taiwan were enrolled in the study from August 2013 to July 2014. The two group baseline patient characteristics were all collected and compared. The trauma patients with suspected pelvic fractures initially stabilized with a pelvic binder had shorter hospital and intensive care unit (ICU) stays. The study group achieved statistically significantly improved survival and lower mean blood transfusion volume and mortality rate, although they were more severe in the trauma score. Conclusions: We recommend prompt pelvic binder use for suspected pelvic injury before definitive imaging is available, as a cervical spine collar is used to protect the cervical spine from further injury prior to definitive identification and characterization of an injury.

## 1. Introduction

Although patients with severe pelvic fractures present many challenges to the trauma team, a correct diagnosis of pelvic injury is crucial since pelvic injuries often occur in conjunction with other life-threatening injuries. However, there is currently no universal consensus on all aspects of the management of pelvic injuries.

Among patients with multiple injuries because of blunt trauma, 5–16% sustain injuries to the pelvic ring, resulting in a mortality rate of 11–54% that is primarily due to hemorrhagic shock [1,2,3]. Therefore, it is important to control associated hemorrhage when managing pelvic fractures. In most trauma units, the initial management of a pelvic fracture is based on the Advanced Trauma Life Support (ATLS) guidelines developed by the American College of Surgeons (ACS) Committee on Trauma, but these guidelines do not contain data or a consensus on a pelvic stabilization method [4]. In theory, the reduction and stabilization of the pelvic ring can decrease bleeding from the fracture site [5], as a reduction of pelvic volume has been shown to reduce the extent of hemorrhage from such injuries [6]. The sooner that bleeding is brought under control, the greater the chance of avoiding the “lethal triad” of hypothermia, coagulopathy, and acidosis secondary to the hypotension and hypoperfusion of tissue [7]. Early pelvic stabilization by external mechanical compression (EMC) with different devices, such as C-clamps, external fixators, and sheets, can reduce pelvic volume and control hemorrhage [8]. However, the use of C-clamps and external fixators is invasive, requires orthopedic expertise and availability, and limits access to the abdomen for exploration, subsequent nursing care, patient positioning, and skin protection. Common noninvasive methods for pelvic stabilization include sheet wrapping and pelvic binders [9].

Pelvic binders have been used increasingly in recent years. Modern binders are light, easily portable, and simple to apply; moreover, they can be used even in conscious patients, thus reducing pain and movement during transfer. Many western paramedical services and military units are required to carry them at the scene of injury. The application of a pelvic binder has become part of the emergency care of all trauma patients with suspected pelvic fractures, in both the pre-hospital environment and emergency department (ED). The present study aimed to assess the effectiveness of the early use of pelvic binders to treat patients with a suspected high risk of pelvic bleeding from blunt force pelvic fractures.

## 2. Materials and Methods

**Figure 1:**
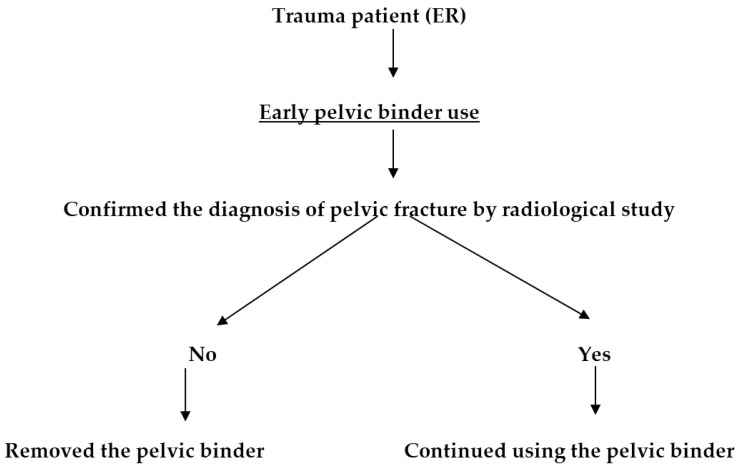
An updated protocol emphasizing the early use of a pelvic binder for trauma patients with suspected pelvic fracture. ER: Emergency Department.

Our hospital is a level I trauma center in Taipei, Taiwan, staffed with in-house attending physicians and equipped with appropriate facilities to manage patients with severe multi-system trauma. This is a retrospective cohort study. The study methods were reviewed and approved by the Institutional Review Board II of the Tri-Service General Hospital, National Defense Medical Center, (TSGHIRB No. 1-103-05-122) and agreed to no informed consent. We enrolled patients (study group) admitted to the ED of Tri-Service General Hospital (TSGH) between August 2013 and July 2014. Enrollment criteria included traumatic injury requiring activation of the trauma team and one of the following risk factors: (1) a loss of consciousness or a Glasgow coma score (GCS) of <13 points; (2) a systolic blood pressure (BP) of <90 mmHg; (3) injury due to falling from a height of 6 m (second floor); (4) injury to multiple vital organs; and/or (5) suspected pelvic injury. From August 2013 to July 2014, patients who met the criteria were enrolled and received early pelvic binder use for the emergency management of suspected pelvic trauma as they arrived at our ED. Patients with trauma injury and any type of pelvic fractures confirmed by radiological imaging (such as pelvic X-ray or computed tomography (CT) scan) in accordance with a new protocol emphasizing the early use of a pelvic binder performed by the ED physicians for trauma patients with suspected pelvic injury were included (Figure 1). Those patients who had no pelvic fractures confirmed by radiological imaging were excluded from the study group and the pelvic binder was immediately removed. Pelvic binders were used to stabilize suspected pelvic fractures in patients with trauma injury in accordance with the ATLS guidelines from the ACS Committee on Trauma. Stabilization of pelvic fractures was achieved by the use of an SAM Pelvic Sling^TM^ II (SAM Medical Products, Wilsonville, OR, USA), which is a commercially available, circumferential pelvic binder made of tightly woven cloth in a ratcheting belt design to achieve uniform, high-pressure, circumferential compression. The SAM Pelvic Sling was applied immediately after a patient’s arrival in our hospital ED and was removed after the possibility of pelvic fracture was excluded by radiological imaging or until a definitive pelvic fracture fixation by an orthopedic surgeon.

In the present study, we compared the characteristics of study group patients with historical control group patients for whom, between January 2011 and July 2013, pelvic binders were only applied after clinical or radiological confirmation of a pelvic fracture. We routinely recorded demographic characteristics, initial vital signs in the ED (blood pressure, respiratory rate, and pulse rate), the revised trauma score (RTS), the injury severity scale (ISS) score, the volume of transfused blood in the first 24 h, intensive care unit (ICU) length of stay (LOS), the percentage of patients in each group with an abbreviated injury score (AIS) of ≤3, and hospital LOS. We also compared the study group with the historical control group about complications related to pelvic binder use, how long to find out about any complications, the duration a patient wore a pelvic binder, the time taken to receive an external fixation, the number of patients receiving pelvic surgery, and the time taken to receive an open reduction and internal fixation (ORIF).

A multivariate logistic regression analysis was used to assess the independent impact of pelvic binder use on treatment outcome adjusted for age, gender, GCS, initial vital signs (blood pressure, respiratory rate, and pulse rate), RTS, ISS, angiography for transcatheter arterial embolization (TAE), AIS, and pelvic fracture types.

The results are presented as mean with standard deviation (SD), proportions, and odds ratios (OR); a probability (*p*) value < 0.05 was considered statistically significant. All statistical analyses were performed using SPSS 13.0 statistical software package for Windows (SPSS, Inc., Chicago, IL, USA).

## 3. Results

In the study group, 56 patients with trauma injury and pelvic fractures confirmed by radiological imaging and who had received early use of a pelvic binder were enrolled. In the historical group, there were 148 patients who suffered from trauma injury and pelvic fractures confirmed by radiological imaging and then received use of a pelvic binder. There were no significant differences in patient age, gender, hospital LOS, ICU LOS, RTS, ISS score, percentage of systolic blood pressure < 90 mmHg, GCS, percentage of AIS ≤ 3, angiography for TAE, type of pelvic fracture, or treatment outcome between groups (Table 1). Patients with suspected pelvic fractures with an initial placement of a pelvic binder achieved significantly improved survival than those for whom a pelvic binder was not initially used, but this tendency did not reach statistical significance. Although there were no statistically significant differences between these two groups, trauma patients with suspected pelvic fractures that were initially stabilized with a pelvic binder had shorter hospital and ICU stays (16.11 ± 12.54 vs. 19.55 ± 26.14 days and 5.33 ± 5.42 vs. 8.36 ± 11.52 days). AIS, hypotension, and fracture classification was more severe in those patients for whom suspected pelvic fractures were initially stabilized with a pelvic binder. However, the average volume of transfused blood in the first 24 h was significantly lower for patients who were initially stabilized with a pelvic binder (2462 ± 2215 mL vs. 4385 ± 3326 mL, respectively; *p* < 0.01).

We also compared the study group with the historical control group about the complications of using a pelvic binder. There were no statistically significant differences between these two groups, but trauma patients with suspected pelvic fractures who were initially stabilized with a pelvic binder had a longer time to find complication (42 ± 8 vs. 57 h; *p* = 0.08) (Table 2).

Multivariate logistic regression revealed that after adjustment for potential confounders, including the percentage of systolic blood pressure < 90 mmHg in the ED, respiration rate at arrival, and volume of transfused blood in the first 24 h, because they reached or were near statistical significance, a univariate analysis showed a tendency of a shorter ICU LOS for the group with suspected pelvic fractures that were initially stabilized with a pelvic binder, but this tendency did not reach statistical significance (OR, 0.95; *p* = 0.269). After adjustment for the influence of confounders, the group with suspected pelvic fractures initially stabilized with a pelvic binder achieved significantly lower mortality in multivariate analysis (OR, 0.00326; *p* = 0.039) (Table 3).

## 4. Discussion

At our hospital, the initial resuscitation, diagnostic evaluation, and management of trauma patients with blunt or penetrating trauma are based on protocols from the ATLS program, established by the ACS Committee on Trauma [4].

Pelvic ring fractures account for approximately 3% of all skeletal fractures [10]. Closed pelvic ring disruptions in patients with multiple injuries are associated with a mortality rate of 10–15%, where those associated with intracranial mass lesions or notable abdominal injuries have mortality rates as high as 50%. Pelvic injuries in particular often occur in conjunction with other life-threatening injuries, among which it is especially important to consider hypotension. In cases of suspected pelvic fracture, it is recommended that ED physicians apply gentle pressure over the iliac wings in a downward and medial fashion to identify laxity and instability. In trauma patients, manual manipulation of the pelvis may be detrimental, as a formed blood clot may dislodge resulting in further hemorrhage. Therefore, this procedure should be performed only once during the physical examination, as testing for pelvic instability can result in further hemorrhage [4]. The results of two retrospective studies showed that the sensitivity of pelvic compression to detect a pelvic fracture was only about 8% [11,12]. Once a pelvic fracture is suspected as the primary source of hemodynamic instability after prompt differentiation from other life-threatening injuries, such as hemothorax, cardiac tamponade, or hemoperitoneum, we always use noninvasive methods for pelvic stabilization, including external fixation, use of a commercially designed pelvis binder, or simple pelvic wrapping with a sheet.

However, the process of differential diagnosis of trauma patients in the ED is time-consuming. The sooner bleeding is controlled, the greater chance of preventing the “lethal triad” of hypothermia, coagulopathy, and acidosis secondary to the hypotension and hypoperfusion of tissues [7,13]. However, a significant proportion of deaths from pelvic fracture are due to exsanguination. The reduction and stabilization of the pelvic ring are presumed to decrease bleeding at the fracture site. Various methods have been described to stabilize the pelvis and reduce pelvic volume [5,14,15]. Closure of the pelvic ring is thought to tamponade bleeding by diminishing the pelvic volume and accelerating the clotting of a pelvic hematoma.

In recent years, the use of a pelvic binder has become widely adopted in resuscitation protocols worldwide and is well-established in many trauma care facilities [16,17]. Chih-Yuan Fu et al. evaluated the use of pelvic compression devices in patients with pelvic fractures who required interhospital transfer, and found a reduction in transfusion requirement, ICU length of stay, and hospital LOS both in stable and unstable fractures [18]. However, Ghaemmaghami et al. demonstrated that early pelvic compression using pelvic binders may have limited use in centers with the availability of angioembolization [19]. Till now, no universal consensus on all aspects of the management of pelvic fracture has been made. Besides, the efficacy of the early use of a pelvic binder in the ED for the management of suspected pelvic trauma remains unclear.

Fracture stabilization decreases pelvic volume, promotes tamponade of venous bleeding, and prevents shifting of the bony elements, which can lead to secondary hemorrhage. The rate of hemorrhage in unstable pelvic fractures ranges from 18 to 62.5%, and venous bleeding is the source of hemorrhage in 80–90% of cases [20,21,22]. The iliolumbar vein was found to be disrupted in 60% of cases with pelvic fractures, accounting for the venous hemorrhage observed in fractures of the sacroiliac portion of the pelvis. Moreover, Baque et al. [23] demonstrated a 20% increase in pelvic volume with a 5-cm pubic diastasis in a cadaver pelvic-fracture model, and Stover et al. [24] demonstrated an increase in pelvic volume of 35–40% with a large 10-cm pubic diastasis, again in a cadaver model.

To our knowledge, the early use of pelvic binders does not reduce pelvic arterial hemorrhage because it may not generate a sufficient tamponade effect deep within the soft pelvic tissues, but it can provide compression and a tamponade effect, which reduces venous hemorrhage [12,25]. Pelvic angiography with embolization is useful to control arterial hemorrhage, but because this procedure controls only arterial hemorrhage, it is beneficial in only 3–10% of patients with pelvic fractures [26,27,28]. The requirement of angioembolization can be predicted by the presence of intravenous contrast extravasation (ICE) on computed tomography (CT), which has a sensitivity of 60–84%, specificity of 85–98%, and positive predictive value of 80%, regardless of hemodynamic status [29,30,31,32]. In fact, the absence of ICE on admission CT is an excellent indicator for excluding the presence of active arterial hemorrhage, and, therefore, the need for angioembolization, with negative predictive values 98.0–99.8% [33,34,35]. However, these examination procedures take so long to confirm a diagnosis of pelvic fracture that they allow many critical patients’ lives to be lost.

Thus, when a pelvis injury is suspected in a hemodynamically unstable patient, physicians should stabilize or “close” the pelvis by securing either a sheet or commercial binder around the fracture, when possible, to reduce pelvic volume and stabilize bone fragments, thereby reducing the risk of major hemorrhage. However, Hedrick-Thompson JK [36] showed that pressure may cause soft tissue or skin damage. Some studies have suggested that a polytrauma patient is likely to be at increased risk of soft-tissue damage due to systemic factors promoting tissue breakdown and trauma-associated local soft tissue injury [37,38]. Knopps et al. recommended that pelvic binders should be used in the short term [39]. In our study, the comparison of these two groups showed no statistical significance in using pelvic binders, but only showed some high risk in wearing a pelvic binder for too long, which may cause skin necrosis. By the way, a pelvic binder should be limited to use for the short term and cushions should be used in the gluteal fold to prevent tissue breakdown.

Pelvic stabilization reportedly maintains and restores mechanical stability to the pelvis and hemodynamic stability to the pelvic fracture before surgical intervention or angiography [40,41]. A pelvic binder is a cost-effective and non-invasive tool and can be used by physicians in an emergency department’s resuscitative period or by an emergency medical technician (EMT) in a pre-hospital situation. It can be the bridge to support hemodynamic unstable patients to receive definitive life-saving procedures. The early use of a pelvic binder can lead to the stabilization of vital parameters within a short period. In addition, the establishment of hybrid operating rooms in recent years has allowed trauma surgeons to perform resuscitation and differential diagnosis more quickly. In this way, we can avoid life-threatening scenarios and save more patients’ lives.

A previous study compared stabilization with a pelvic binder to emergency pelvic external fixation in 186 patients and found that the requirement for transfusion was significantly lower in the study group at 24 h (4.9 vs. 17.1 U; *p* < 0.0001) and 48 h (6.0 vs. 18.6 U; *p* < 0.0001). Moreover, the length of hospital stay (16.5 vs. 24.4 days; *p* = 0.03) and mortality (26% vs. 37% for pelvic orthotic device and emergency pelvic fixation, respectively; *p* = 0.11) was reduced in the binder group, although this difference was not statistically significant [2].

In our study, we found that a transfusion requirement was significantly reduced in patients receiving prompt stabilization with use of a pelvic binder. The length of ICU stay also showed a decreasing tendency, but did not reach statistical significance. Although none of these differences were statistically significant, it is possible that patients may have experienced worse outcomes had it not been for the early use of a pelvic binder and the study group is too small to reach statistical significance.

### Limitation

There were a few potential limitations to our study. It was a single-center experience, and may reflect local patient characteristics. As with most retrospective studies, unmeasured or unknown variables may be responsible for the effects seen, and the subsequent conclusions formulated. We wish that, in the future, many investigations would be available with evidence to support our conclusions.

## 5. Conclusions

Because of the ease of application, relatively inexpensive cost, low potential for complications, and benefit to pelvic stability, we recommend the early use of a pelvic binder if pelvic injury is suspected before definitive imaging is available, in the same way as a cervical spine collar is used to protect the cervical spine from further injury prior to the definitive identification and characterization of an injury.

## Figures and Tables

**Table 1:** Baseline patient characteristics.

Variable	Before Study Group (n = 148)	Study Group (n = 56)	*p*-Value
	Mean (Standard Deviation)	Mean (Standard Deviation)	
Age	45.14 (20.96)	46.36 (21.07)	0.711
Gender (M/F)	1.11 (78/70)	0.86 (26/30)	0.520
Hospital_LOS	19.55 (26.14)	16.11 (12.54)	0.346
ICU_LOS	8.36 (11.52)	5.33 (5.42)	0.252
RTS	7.26 (1.89)	7.12 (1.62)	0.609
ISS	15.80 (12.02)	16.91 (13.77)	0.571
Hypotension (systolic blood pressure ≤ 90), n (%)	12 (8.1%)	10 (17.6%)	0.09
respiration	18.26 (3.66)	19.63 (2.32)	0.043
GCS	13.86 (3.30)	13.66 (3.20)	0.704
Blood transfusion (mL)	4385 (3326)	2462 (2215)	0.009
Abbreviated injury score, n (%)			0.365
≤3	114 (77.0%)	39 (69.6%)	
>3	34 (23.0%)	17 (30.4%)	
Associated injury, n (%)			0.732
Yes	42 (28.38%)	18 (32.14%)	
No	106 (71.62%)	38 (67.86%)	
Angiography for TAE ^a^, n (%)			0.878
Yes	2 (1.35%)	1 (1.79%)	
No	146 (98.65%)	55 (98.21%)	
Outcome, n (%)			0.785
Survive	131 (88.51%)	51 (91.07%)	
Mortality	17 (11.49%)	5 (8.93%)	
Fracture classification ^b^, n (%)			
L	124 (83.8%)	45 (80.4%)	0.710
A	21 (14.2%)	9 (16.1%)	0.907
V	3 (2.0%)	2 (3.6%)	0.617
Complication related to use pelvic binder (skin necrosis, soft tissue damage or ischemic change)	2 (1.35%)	1 (1.79%)	0.731

Values are presented as means and SD unless otherwise indicated. ^a^ transcatheter arterial embolization (TAE) was specific to the hemostasis of pelvic fracture-related retroperitoneal hemorrhage. ^b^ fracture classification: L (Lateral compression), A (Anterior posterior compression), V (Vertical shear). Abbreviations: LOS (length of stay), ICU (intensive care unit), RTS (revised trauma score), ISS (injury severity scale), GCS (Glasgow coma score).

**Table 2:** A comparison of the study group with the historical control group of using pelvic binders.

Parameter	Historical Control Group (n = 148)	Study Group (n = 56)	*p*-Value
Complication related to use pelvic binder (No.)	2 (1.35%)	1 (1.79%)	0.731
skin necrosis	2	1	
soft tissue damage	0	0	
ischemic change	0	0	
* Time to find complications (Hours)	42 ± 8	57 ± 7	0.08
* Duration of using pelvic binder (Days)	2.6 ± 0.8	2.9 ± 0.7	0.792
* Time to receive external fixation (Days)	2.1 ± 1.1	2.7 ± 0.9	0.478
No. of receiving pelvic surgery	58	18	0.882
* Time to receive ORIF (Days)	6.8 ± 1.3	7.1 ± 1.5	0.897

ORIF: open reduction and internal fixation. * Mean ± SD.

**Table 3:** Logistic regression analysis of risk factors.

Variable	Univariate OR (95% CI)	*p*-Value	Multivariate OR (95% CI)	*p*-Value
ICU_LOS	0.95 (0.87–1.04)	0.269	0.77 (0.51–1.17)	0.219
Result (died vs. nondied)	0.76 (0.27–2.16)	0.600	0.00326 (0.00001–0.73888)	0.039

OR—odds ratio; CI—confidence interval. Logistic regression was used to adjust for age, gender, systolic blood pressure, prerespiration, respiration, ISS, morbidity, angiography for TAE, AIS, and fracture classification.

## References

[1] Giannoudis P.V., Grotz M.R., Tzioupis C., Dinopoulos H., Wells G.E., Bouamra O., Lecky F. (2007). Prevalence of pelvic fractures, associated injuries, and mortality: The United Kingdom perspective. J. Trauma.

[2] Croce M.A., Magnotti L.J., Savage S.A., Wood G.W., Fabian T.C. (2007). Emergent pelvic fixation in patients with exsanguinating pelvic fractures. J. Am. Coll. Surg..

[3] Heckbert S.R., Vedder N.B., Hoffman W., Winn R.K., Hudson L.D., Jurkovich G.J., Copass M.K., Harlan J.M., Rice C.L., Maier R.V. (1998). Outcome after hemorrhagic shock in trauma patients. J. Trauma.

[4] Committee on Trauma, American College of Surgeons (2012). Advanced Trauma Life Support.

[5] DeAngelis N.A., Wixted J.J., Drew J., Eskander M.S., Eskander J.P., French B.G. (2008). Use of the trauma pelvic orthotic device (T-POD) for provisional stabilisation of anterior-posterior compression type pelvic fractures: A cadaveric study. Injury.

[6] Moreno C., Moore E.E., Rosenberger A., Cleveland H.C. (1986). Haemorrhage associated with major pelvic fracture. J. Trauma.

[7] Van Vugt A.B., van Kampen A. (2006). An unstable pelvic ring. The killing fracture. J. Bone Jt. Surg..

[8] Pizanis A., Pohlemann T., Burkhardt M., Aqheyev E., Holstein J.H. (2013). Emergency stabilization of the pelvic ring: Clinical comparison between three different techniques. Injury.

[9] Krieg J.C., Mohr M., Ellis T.J., Simpson T.S., Madey S.M., Bottlang M. (2005). Emergent stabilization of pelvic ring injuries by controlled circumferential compression: A clinical trial. J. Trauma.

[10] Grotz M.R., Allami M.K., Harwood P., Ape H.C., Krettek C., Giannoudis P.V. (2005). Open pelvic fractures: Epidemiology, current concepts of management and outcome. Injury.

[11] Shlamovitz G.Z., Mower W.R., Bergman J., Chuang K.R., Crisp J., Hardy D., Sargent M., Shroff S.D., Snyder E., Morgan M.T. (2009). How (un)useful is the pelvic ring stability examination in diagnosing mechanically unstable pelvic fractures in blunt trauma patients?. J. Trauma.

[12] Fu C.Y., Wu S.C., Chen R.J., Wang Y.C., Chung P.K., Yeh C.C., Huang H.C. (2009). Evaluation of pelvic fracture stability and the need for angioembolization: Pelvic instabilities on plain film have an increased probability of requiring angioembolization. Am. J. Emerg. Med..

[13] Eddy V.A., Morris J.A., Cullinane D.C. (2000). Hypothermia, coagulopathy, and acidosis. Surg. Clin. N. Am..

[14] Starr A.J., Griffin M.A. Pelvic ring disruptions: Mechanism, fracture pattern, morbidity and mortality: An analysis of 325 patients. Proceedings of the Orthopaedic Trauma Association 16th Annual Meeting.

[15] Gylling S.F., Ward R.E., Holcroft J.W., Bray T.J., Chapman M.W. (1985). Immediate external fixation of unstable pelvic fractures. Am. J. Surg..

[16] Eastridge B.J., Starr A., Minei J.P., O’Keefe G.E., Scalea T.M. (2002). The importance of fracture pattern in guiding therapeutic decision-making in patients with hemorrhagic shock and pelvic ring disruptions. J. Trauma.

[17] Kortbeek J.B., Al Turki S.A., Ali J. (2008). Advanced trauma Life Support, 8th edition, the evidence for change. J. Trauma.

[18] Fu C.Y., Wu Y.T., Liao C.H., Kang S.C., Wang S.Y., Hsu Y.P., Lin B.C., Yuan K.C., Kuo I.M., Ouyang C.H. (2013). Pelvic circumferential compression devices benefit patients with pelvic fractures who need transfers. Am. J. Emerg. Med..

[19] Ghaemmaghami V., Sperry J., Gunst M., Friese R., Starr A., Frankel H., Gentilello L.M., Shafi S. (2007). Effects of early use of external pelvic compression on transfusion requirements and mortality in pelvic fractures. Am. J. Surg..

[20] Gänsslen A., Giannoudis P., Pape H.C. (2003). Hemorrhage in pelvic fracture: Who needs angiography?. Curr. Opin. Crit. Care.

[21] Cole P.A. (2003). What’s new in orthopaedic trauma. J. Bone Jt. Surg. Am..

[22] Fu C.Y., Wang Y.C., Wu S.C., Chen Y.F., Chen R.J., Hsieh C.H., Huang H.C., Huang J.C., Lu C.W., Huang Y.C. (2012). Higher glucose on admission is associated with need for angioembolization in stable pelvic fracture. Am. J. Emerg. Med..

[23] Baque P., Trojani C., Delotte J., Séjor E., Senni-Buratti M., de Baqué F., Bourgeon A. (2005). Anatomical consequences of “open-book” pelvic ring disruption: A cadaver experimental study. Surg. Radiol. Anat..

[24] Stover M.D., Summers H.D., Ghanayem A.J., Wilber J.H. (2006). Three-dimensional analysis of pelvic volume in an unstable pelvic fracture. J. Trauma.

[25] Hamill J., Holden A., Paice R., Civil I. (2000). Pelvic fracture pattern predicts pelvic arterial haemorrhage. Aust. N. Z. J. Surg..

[26] Totterman A., Dormagen J., Madsen J.E., Klow N.E., Skaga N.O., Roise O. (2006). A protocol for angiographic embolization in exsanguinating pelvic trauma: A report on 31 patients. Acta Orthop..

[27] Miller P.R., Moore P.S., Mansell E., Meredith J.W., Chang M.C. (2003). External fixation or arteriogram in bleeding pelvic fracture: Initial therapy guided by markers of arterial hemorrhage. J. Trauma.

[28] Cook R.E., Keating J.F., Gillespie I. (2002). The role of angiography in the management of haemorrhage from major fractures of the pelvis. J. Bone Jt. Surg..

[29] Fangio P., Asehnoune K., Edouard A., Smail N., Benhamou D. (2005). Early embolization and vasopressor administration for management of lifethreatening hemorrhage from pelvic fracture. J. Trauma.

[30] Gourlay D., Hoffer E., Routt M., Bulger E. (2005). Pelvic angiography for recurrent traumatic pelvic arterial hemorrhage. J. Trauma.

[31] Fang J.F., Shih L.Y., Wong Y.C., Lin B.C., Hsu Y.P. (2009). Repeat transcatheter arterial embolization for the management of pelvic arterial hemorrhage. J. Trauma.

[32] Stephen D.J.G., Kreder H.J., Day A.C., McKee M.D., Schemitsch E.H., ElMaraghy A., Hamilton P., McLellan B. (1999). Early detection of arterial bleeding in acute pelvic trauma. J. Trauma.

[33] Pereira S.J., O’Brien D.P., Luchette F.A., Choe K.A., Lim E., Davis K., Hurst J.M., Johannigman J.A., Frame S.B. (2000). Dynamic helical computed tomography scan accurately detects hemorrhage in patients with pelvic fracture. Surgery.

[34] Ryan M.F., Hamilton P.A., Chu P., Hanaghan J. (2004). Active extravasation of arterial contrast agent on post-traumatic abdominal computed tomography. Can. Assoc. Radiol. J..

[35] Evers B.M., Cryer H.M., Miller F.B. (1989). Pelvic fracture hemorrhage: Priorities in management. Arch. Surg..

[36] Hedrick-Thompson J.K. (1992). A review of pressure reduction device studies. J. Vasc. Nurs..

[37] Lerner A., Fodor L., Keren Y., Horesh Z., Soudry M. (2008). External fixation for temporary stabilization and wound management of an open pelvic ring injury with extensive soft tissue damage: Case report and review of the literature. J. Trauma.

[38] Philips T.J., Jeffcote B., Collopy D. (2008). Bilateral Morel-Lavallee lesions after complex pelvic trauma: A case report. J. Trauma.

[39] Knops S.P., Van Lieshout E.M., Spanjersberg W.R., Patka P., Schipper I.B. (2011). Randomised Clinical Trial Comparing Pressure Characteristics of Pelvic Circumferential Compression Devices in Healthy Volunteers. Injury.

[40] Biffl W.L., Smith W., Moore E.E., Gonzalez R.J., Morgan S.J., Hennessey T., Offner P.J., Ray C.E., Franciose R.J., Burch J.M. (2001). Evolution of a multidisciplinary clinical pathway for the management of unstable patients with pelvic fracture. Ann. Surg..

[41] Heetveld M.J., Harris I., Schlaphoff G., Sugrue M. (2004). Guidelines for the management of haemodynamically unstable pelvic fracture patients. ANZ J. Surg..

