# Correction: Hsu et al. Effect of Early Pelvic Binder Use in the Emergency Management of Suspected Pelvic Trauma: A Retrospective Cohort Study. *Int. J. Environ. Res. Public Health* 2017, *14*, 1217

**DOI:** 10.3390/ijerph19116654

**Published:** 2022-05-30

**Authors:** Sheng-Der Hsu, Cheng-Jueng Chen, Yu-Ching Chou, Sheng-Hao Wang, De-Chuan Chan

**Affiliations:** 1 Division of Traumatic Surgery, Department of Surgery, Tri-Service General Hospital, National Defense Medical Center, Taipei 11486, Taiwan; 2 Division of General Surgery, Department of Surgery, Tri-Service General Hospital, National Defense Medical Center, Taipei 11486, Taiwan; doc20227@ndmctsgh.edu.tw (C.-J.C.); doc20230@ndmctsgh.edu.tw (D.-C.C.); 3 School of Public Health, National Defense Medical Center, Taipei 11490, Taiwan; trishow@ndmctsgh.edu.tw; 4 Division of Orthopedic Surgery, Tri-Service General Hospital, National Defense Medical Center, Taipei 11486, Taiwan; doc20361@ndmctsgh.edu.tw

**Text Correction** 

This corrects the article “Effect of Early Pelvic Binder Use in the Emergency Management of Suspected Pelvic Trauma: A Retrospective Cohort Study” in volume 14 on pages 4 and 5 of 9.

The authors wish to add the following amendments and corrections to their paper published in IJERPH [1].

A correction has been made to Section 3, third paragraph:

The sentences “but this tendency did not reach statistical significance (OR, 0.9; *p* < 0.302). After adjustment for the influence of confounders, the group with suspected pelvic fractures that were initially stabilized with a pelvic binder achieved significantly lower mortality (OR, 0.04; *p* < 0.030) in the univariate analysis, and also in the multivariate analysis (OR, 0.00326; *p* < 0.039) (Table 3).” should be corrected to “but this tendency did not reach statistical significance (OR, 0.95; *p* = 0.269). After adjustment for the influence of confounders, the group with suspected pelvic fractures initially stabilized with a pelvic binder achieved significantly lower mortality in multivariate analysis (OR, 0.00326; *p* = 0.039) (Table 3).”.

**Error in Table **

On page 5 of 9, Table 3, the univariate OR (95% CI) and *p*-value of the “Logistic regression analysis of risk factors” column were incorrectly calculated. We have corrected this critical error. The updated Table 3 should be as follows:

**Table 3:** Logistic regression analysis of risk factors.

Variable	Univariate OR (95% CI)	*p*-Value	Multivariate OR (95% CI)	*p*-Value
ICU_LOS	0.95 (0.87–1.04)	0.269	0.77 (0.51–1.17)	0.219
Result (died vs. nondied)	0.76 (0.27–2.16)	0.600	0.00326 (0.00001–0.73888)	0.039

OR—odds ratio; CI—confidence interval. Logistic regression was used to adjust for age, gender, systolic blood pressure, prerespiration, respiration, ISS, morbidity, angiography for TAE, AIS, and fracture classification.

The authors apologize for any inconvenience caused and state that the scientific conclusions are unaffected. The original article has been updated.
